# Is the Philippines ready for HIV self-testing?

**DOI:** 10.1186/s12889-019-8063-8

**Published:** 2020-01-09

**Authors:** Jesal Gohil, Emmanuel S. Baja, Tyrone Reden Sy, Ernest Genesis Guevara, Charlotte Hemingway, Paul Mark B. Medina, Leila Coppens, Godofreda V. Dalmacion, Miriam Taegtmeyer

**Affiliations:** 1 Department of Medicine, The Royal London Hospital, London, UK; 2 Institute of Clinical Epidemiology, National Institutes of Health, University of the Philippines-Manila, Manila, Philippines; 3 Department of Clinical Epidemiology, College of Medicine, University of the Philippines-Manila, Manila, Philippines; 4 Department of International Public Health, Liverpool School of Tropical Medicine, Pembroke Palace Liverpool, Liverpool, L3 5QA UK; 5 Department of Biochemistry and Molecular Biology, College of Medicine, University of the Philippines-Manila, Manila, Philippines; 6 World Health Organization (WHO) Philippines, Manila, Philippines; 7 Department of Pharmacology and Toxicology, College of Medicine, University of the Philippines-Manila, Manila, Philippines

**Keywords:** HIV self-testing, Philippines, Men who have sex with men (MSM), Transgender women, TGW, Regulation, Policy

## Abstract

**Background:**

The Philippines is facing a rapidly rising HIV epidemic among young men who have sex with men (MSM). Testing rates among young populations is poor. HIV self-testing (HIVST) is a promising strategy to address this testing gap. The study’s purpose was to explore the perceived acceptability, feasibility and programmatic challenges of HIVST among key informants and target users.

**Method:**

A qualitative study involving semi-structured interviews and focus group discussions (FGD). We interviewed 15 key informants involved with HIV testing programs or policies and 42 target users in six FGD in Metro Manila. We held separate discussions with high socio-economic MSM (*n* = 12), urban poor MSM (*n* = 15) and transgender women (TGW) (n = 15). Results were analysed using a thematic framework approach.

**Results:**

MSM and TGW welcomed the convenience and privacy HIVST could provide. They preferred an inexpensive accurate blood-based kit attained from reputable sites. Key informants at national and local level equally welcomed HIVST but identified a number of policy and regulatory issues. Both groups articulated the challenge of enrolling those who test reactive using HIVST to further testing and treatment in an environment characterised by acute stigma around HIV.

**Conclusions:**

HIVST was found to be highly acceptable to target users and was welcomed as an additional testing approach at national level. Strategic alliances are now needed between stakeholders to proactively deliver a patient-centred HIVST program that could provide an effective, safe means of increasing testing coverage in this escalating context.

## Background

The Philippines is experiencing the most rapidly escalating HIV epidemic in the world [1]. Between 2010 and 2017 there has been a 174% increase of the HIV incidence [2]. A concentrated epidemic exists among men who have sex with men (MSM) and transgender women (TGW), with eight regions having a prevalence above 5% in these groups [3]. Specific data for TGW are limited [4], although a cross-sectional study in Cebu City in 2015 showed a 11.8% HIV prevalence in this group [5]. There is currently a large unmet need for HIV testing among both MSM and TGW. An estimated 63% of MSM and TGW living with HIV diagnosed; disaggregating further into age groups surfaces the disadvantageous position of young key populations with 4% of the estimated people living with HIV aged 15 to 19 diagnosed, and 26% of 20 to 24-year olds [6].

The restrictive legal and regulatory framework, has contributed to low HIV testing uptake, low condom use and poor linkage to care in the Philippines [7, 8], with a disproportionate effect on MSM and TGW below 18. Current national policy demands that HIV testing be carried out by a laboratory technologist specifically trained for HIV testing and that only individuals 18 or older may buy condoms or consent for testing (with minors requiring the consent of a parent or guardian) (Republic Act No.8504). Many local governments have enacted ordinances whereby condoms are seen as proofs of prostitution, an illegal activity. Overall the level of provider initiated counselling is low except among overseas workers and entertainment workers based in establishments.

Facility based testing is the main model of proving HTS in the Philippines [1]. HIV testing can only occur in a centre which has received accreditation by the Department of Health (Republic Act No.8504). A medical technologist that has been certified with HIV proficiency testing, is only allowed to conduct a test. They are only able to use diagnostic kits that have been registered by the FDA. HIV testing, consultation and treatment is free in the Philippines.

Community-based screening, involving HIV rapid diagnostic screening tests performed by trained lay providers, has been piloted in Metro Manila, Cebu and Davao to increase uptake of HIV testing amongst key populations. These outreach efforts have contributed to increased numbers of key populations knowing their HIV status. However, the overall impact of rapid testing on the uptake of testing and on linkage to care and treatment is likely to fall far short of what is required [9, 10]. According to the last Integrated Behavioural and Serological Survey conducted in 35 sites, only 16% of MSM and TGW respondents had an HIV test and received results during the past 12 months [5].

HIV self-testing (HIVST) is an innovative strategy recommended by the WHO [11, 12] that has been successful in increasing testing uptake among key populations in China [13], Hong Kong [14] and Vietnam [15]. It involves individuals conducting a rapid diagnostic HIV test using their own blood or oral fluid specimen, and interpreting the result. HIVST provides individuals with an accurate, convenient test that can assure privacy and confidentiality, overcoming traditional barriers to facility-based testing among key populations [12, 16, 17]. Available studies from Asia, have shown HIVST to be acceptable among MSM and TGW [18–21].

The Philippines does not have a HIVST policy or any formal regulation of HIVST kits [22]. However unregulated HIVST kits can be purchased through the internet [11, 12]. The National Reference Laboratory is currently validating one assay.

To date very little research has been conducted in the Philippines regarding HIVST. In particular if HIVST is an acceptable and feasible strategy to increase uptake of HIV testing among key populations. Local research is required to inform national policies and procedures for HIVST. To address this knowledge gap, we aimed to explore attitudes and perceptions of HIVST amongst key informants, TGW and MSM. Understanding their perceptions would show if key populations would consider HIVST, and reveal variables that may pose programmatic challenges for policies, regulation and culture.

## Methods

The study was conducted in Metro Manila, Philippines between May and June 2017. We used qualitative methods to explore perceptions of MSM, TGW, service providers and policy makers for HIVST, and to understand the potential barriers, opportunities and challenges in policy and regulation.

We used semi-structured interviews with key informants due to its flexible nature that allowed the core and emerging themes to be covered together. FGDs with MSM and TGW allowed debate to occur regarding HIVST amongst the high-risk populations and enabled the triangulation of results.

### Participants

Fifteen key informant interviews were conducted. We purposively selected key informants who were involved with designing and implementing programs or policies relating to HIV testing at a local or national level (Table 1).

**Table 1:** Characteristics of Key-Informants and FGD participants

Key-Informants and FGD participants	Number of participants
Program implementers	4 (3 females, 1 male)
Doctors and academics	3 (1 male, 2 female)
Members of the national or local reference laboratories for HIV	1 (female)
Members of non-governmental organisations	2 (1 male, 1 female)
Service providers	5 (5 male)
MSM high socio-economic group 1	6
MSM high socio-economic group 2	6
MSM urban poor areas	7
MSM urban poor areas	8
TGW group 1	8
TGW group 2	7

Key informants in this study included professions such as program implementers, non-governmental organisations, doctors and academics, service providers and members associated with reference laboratories for HIV.

The initial list of contacts was generated by consultation with the research team at the University of Philippines and their contact directory. Snowball sampling identified additional key informants that were contacted by email or telephone. There was a 94% response rate. Forty-two participants were involved in six FGDs with MSM and TGW aged over 18 years (Table 1). Four FGDs involved men who identified themselves as a man who has sex with men (27 participants). Two of these FGDs with MSM involved individuals from urban poor areas in order to capture a range of perspectives. Two FGD involved TGW who are biologically male and identified themselves as female (15 participants). Recruitment of MSM from high socio-economic groups was through gay social media applications Growler, Grindr and Planet Romeo. TGW and MSM from urban poor areas were recruited through advertising by community organisations and peer outreach workers.

### Data collection

Semi-structured interviews with key informants was administered in English, at a place chosen by the participant and lasted up to an hour. The topic guide included questions which focused on the current policies relating to HIV testing; their attitudes of HIVST; target populations; the benefits and harms of HIVST; the characteristics of HIVST kits; the perceived regulation and monitoring processes and the challenges of implementing HIVST in the country.

FGDs with MSM and TGW involved between 6 and 8 participants each, lasted up to 2 h and were conducted by trained facilitators in Tagalog or English. Prior to the discussion participants were shown a video demonstrating oral (OraQuick) and blood kits (BioSURE) suitable and approved for HIVST use in other contexts. The topic guide for the discussion included: questions about their opinion on HIV services in the Philippines; the characteristics and procedure of HIVST; their concerns of HIVST; the costs of a kit; the distribution of HIVST kits; counselling services; and the linkage to health care following a HIV self-test.

All interviews were audio-taped, transcribed and discussed amongst the research team post-interview to identify emerging themes and allow topic guides to be revised.

### Data analysis

The analysis was conducted using the thematic framework approach. The first author sorted and coded emerging themes manually. A selection of transcripts were reviewed by a second researcher for the purposes of quality assurance. An analytical coding framework was devised from the transcripts and agreed by the two researchers. Transcripts of the audio recording were indexed utilising an analytical framework. Summarised data were inputted within a framework matrix, followed by in-depth analysis and interpretation.

## Results

MSM and TGW welcomed the idea of introducing HIVST, especially blood-based diagnostic kits, accessed via reputable outlets. They liked the convenience and confidentiality of HIVST in a stigmatised context. Key informants shared enthusiasm for HIVST but also raised concerns regarding antecedents within the health, regulatory and legal system that were at odds with the beneficiaries’ desire for convenience and confidential HIV testing in a stigmatised context. In addition key informants held different views on priorities to address this urgent HIV epidemic.

### HIVST: big benefits and high demand from target users and key informants

HIVST availability was considered by all participants as being a beneficial addition to the HIV testing strategy in the Philippines, particularly in reaching high-risk groups such as young MSM. All key informants stated a major benefit for HIVST would be increased access to testing.

*“it [HIVST] will increase access to HIV testing and other populations that are not reached through our routine testing … ..because of the stigma, discrimination that is still experienced in this country” Laboratory participant*

A majority of MSM and TGW showed enthusiasm and willingness to perform HIVST in the future after watching a video of the testing procedure.

The appealing factors for HIVST in both groups included awareness of HIV status in a confidential, private and convenient manner in comparison to current facility testing, an opinion shared by most key informants.

*“For me, it’s just for myself whether I’m HIV positive or not, nobody else will know if you’re positive” MSM FGD high socio-economic status*

*“People don’t want to go to clinics, it is stigmatising. They don’t want to be seen in those clinics, they may know someone in the facility, or in the area of the facility. They don’t want to get judged” Male Service provider*

MSM and TGW highlighted that self-testing would be empowering, and allow individuals to take responsibility of their own health.

*“It [HIVST] would be easier, you can do it anywhere, anytime, you're at home, in your room, in the toilet, you won't be hassled to go to the clinic, and wait there” TGW FGD 1*

MSM and TGW showed interest in partner testing, and felt HIVST could lead to a shift in relationship dynamics, with increased decision-making regarding sexual intercourse. TGW believed HIVST could make their relationships stronger by increasing mutual trust.

Key informants felt that HIVST could reach new and previous testers. Yet most key informants felt currently HIVST would mostly benefit those with good knowledge and previous testing experience. Doctors and service providers viewed educating the public on HIV as an essential pre-requisite to create demand for a HIVST program and thus reaching new testers. However this would be challenging within the religious and conservative culture present in the Philippines.

*“The most beneficial [group are those] who have their test before, because they have been orientated with HIV 101, they will know the consequences of the test if they ever are positive” Male Doctor*

*“To sustain the use of a HIV self-test, and sustain its market ability, people should understand what the benefits are of using this and this is just not done through marketing, but really making people aware of what HIV is” Female NGO representative*

Concerns were expressed regarding the current knowledge of HIV amongst the public. This was reflected in the varying knowledge of HIV amongst FGD participants as some MSM from urban poor areas showed poor knowledge, requiring misconceptions to be corrected.

*“There are no medicines for HIV, right?” MSM FGD 1 urban poor area*

### Desirable characteristics of HIVST kits and access

Over half of MSM and TGW participants preferred blood-based to oral fluid HIVST kits as they felt it would be more accurate.

*“Blood is better, because you'll know if you have HIV, because it's blood-to-blood”* TGW FGD 1

They felt the kit should have instructions in Tagalog, access to a video demonstration and telephone hotline to aid those with literary difficulties.

All key informants, MSM and TGW felt that a high price would restrict access of HIVST, but this was especially so among participants from poor urban areas. MSM participants from low socio-economic groups were willing to pay up to 200 pesos. Whilst MSM from middle to high economic groups and TGW suggested paying between 50 and 875 pesos and 300 pesos respectively.

### Distribution and linkage to care

A range of options for distribution were mentioned by key informants, MSM and TGW regarding the most suitable avenue for acquiring HIVST kits. Popular options amongst all participants included clinics, community sites and pharmacies. These sites were favoured by MSM and TGW as they were considered trustworthy. Online purchase was favoured by 1 MSM and 1 TGW due to the convenience of buying the product and its delivery, which would eliminate the stigma. However key informants and most MSM and TGW disagreed, and were concerned about fake kits being sold or being discovered by family members. Two program implementers argued for multiple avenues for distribution such as vending machines.

*“Self-testing should be real self-testing, there should be no human-human interaction to get that kit” Male program implementer*

Linkage to HIV services following a reactive result was a frequent concern raised by all participants. All groups emphasised the need for clear information to be included with HIVST kits regarding the processes a tester can expect after a reactive test result. This included the requirement for a confirmatory test, accuracy of test kits, and the location of HIV services.

*“So self-testing is ok, but if I turn out reactive, where do I ask for help?” MSM FGD 1 urban poor area*

*“My predicament about the support, you really cannot separate testing and follow-up, treatment because it does not end from testing yourself. That's why I wanted to make sure that if you're doing it, you have an available support” MSM high socio-economic status.*

*“They should be familiar with what they should do, once the results are positive, because you cannot stop at just testing, and not knowing what to do afterwards” Male Doctor*

*“There is a problem of that linkage to care, to me is also very important, because you don’t want them to test and then nothing happens” Female program Implementer*

### Regulation, readiness and priority

**Fig. 1:**
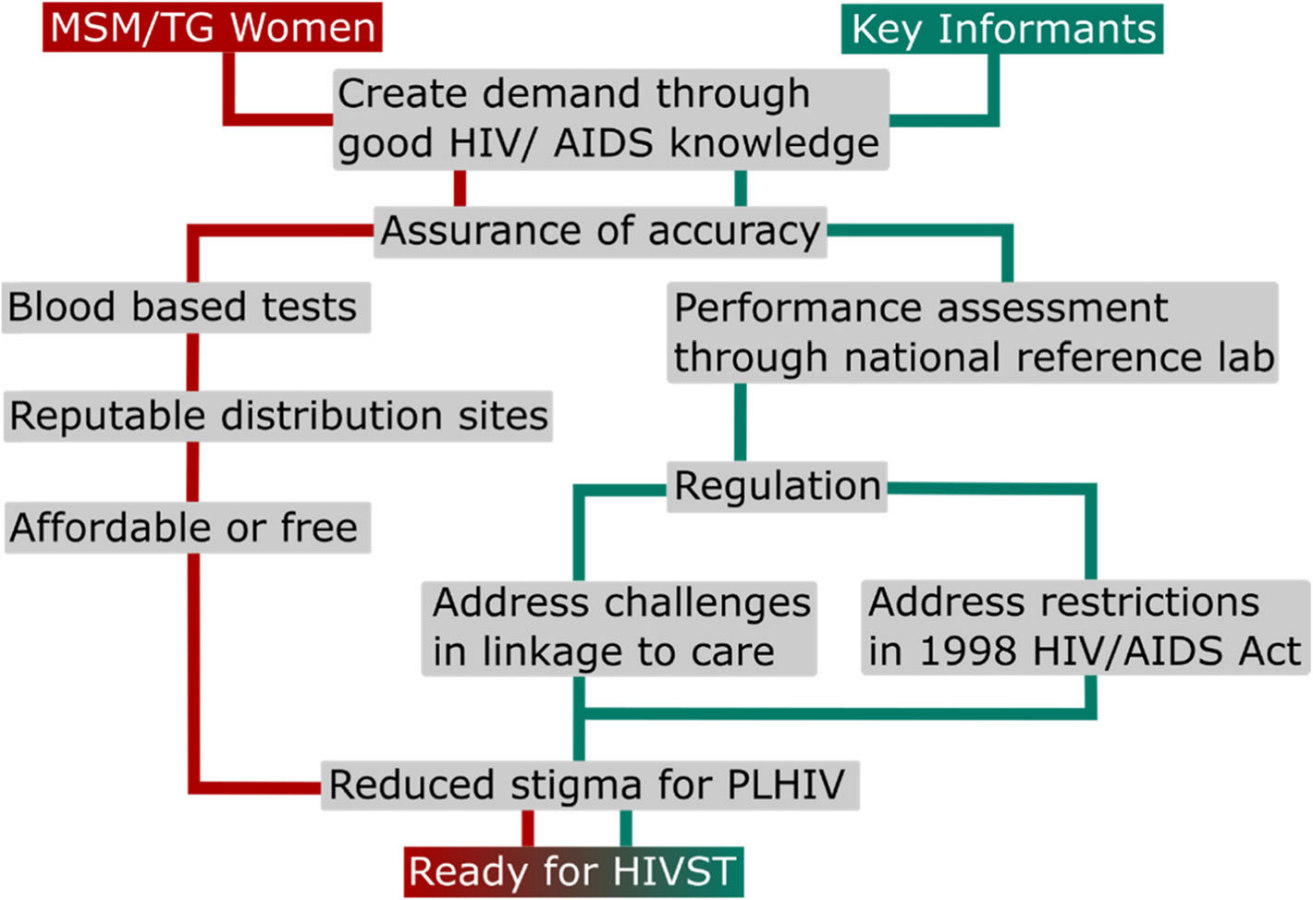
Programmatic considerations for HIVST implementation in the Philippines

Key informants recognised addressing the urgency of the epidemic and were enthusiastic about HIVST, however they felt regulatory, policy and the current health system required changes prior to its implementation (Fig. 1).

All key informants stated that HIVST should be regulated, and identified the FDA as the organisation to oversee this. Regulation was considered essential to ensure accurate test kits were available to the public. The National Laboratory was also identified to be responsible for conducting a performance assessment of HIVST kits. Those which pass the accepted in-country threshold for sensitivity and specificity would be recommended to the FDA.

*“The accuracy of performing the test, and then how they interpret it because the quality of testing is very important because getting a false positive or false negative result might have a tremendous implication for the patients, so it’s very important to get an accurate test” Male Doctor*.

Knowledge regarding the use of HIVST in other contexts was evident amongst all key informants. Most key informants were also aware of HIVST kits being available via the internet. Two MSM participants had prior knowledge of HIVST, and its availability via the internet and also stores within the city.

*“The prevention of abuse of sales of unregistered kits, that is my concern and that is my reservation for HIV self-testing” Participant from HIV reference laboratory*

Key informants found there to be no current policy which supported HIVST in the Philippines. They identified two sections of the Republic Act No.8504 (HIV/AIDS Prevention and Control Act of 1998) which would be a challenge for implementing self-testing, namely pre and post-test counselling by an accredited counsellor, as well as the requirement that HIV testing can only be conducted by a medical technologist. They were however optimistic that the promotion of HIVST as a screening program as opposed to testing, could lift these restrictions. In the Philippines, the testing guidelines from 2017 draw a distinction between screening and testing. While testing is only performed by trained medical technologist, screening is seen as an additional procedure prior to testing that can be performed by lay trained provider, midwives, nurses and physician using rapid diagnostic tests.

Both key informants, MSM and TGW highlighted counselling as important, especially for first-time testers. They recognised that counselling could take several modalities including remote counselling. Peer counsellors and telephone services were popular choices.

*“If you get this test result and then you are not properly counselled, you don’t know where to go, what does it actually mean, then you can end up with a lot of people depressed, angry at the world, so it can cause more harm than good, without the proper set of interventions that go with the introduction of self-testing” Male NGO representative*

Although all key informants were supportive of HIVST, views differed as to the priority for implementation in the Philippines. Some service providers stated this as a high priority program, to provide new tools to fight the growing HIV epidemic. Whilst a few participants stressed strengthening the current health system in terms of HIV testing, linkage to care, increasing ART coverage and reducing loss to follow-up.

*“I think in the Philippines there are more systemic testing issues, and the testing system, that if they were changed and addressed, they would make much more of a change in terms of testing coverage, rather than, more than the introduction of self-testing” Female program implementer.*

## Discussion

Our findings demonstrate that all key informants, and a majority of MSM and TGW in this study are supportive of an HIVST approach being introduced in the Philippines and welcomed the confidentiality, convenience and flexibility, particularly if kits could be available at low or no cost. Despite expressing enthusiasm key informants expressed some concerns centred upon: the legal and policy environment; low rates of linkage and limited available treatment in the existing health system. Some participants therefore felt improving these aspects of the current health system were more of a priority than the introduction of HIVST.

A HIVST strategy that is patient-centred and addresses the concerns of hidden populations is more likely to be relevant in the Philippines context. This includes offering an array of options that will satisfy the different users’ preferences such as the site of distribution, the cost of the kit to the user, the type of kit, and ensuring linkage to care. Whilst stigma is a barrier to HIV testing in general the converse is also true that the convenience and confidentiality provided by HIVST may make it an attractive approach to programmers. Furthermore individuals who test negative have the potential to be linked to other HIV prevention methods such as pre-exposure prophylaxis (PrEP) which is available in Manilla.

Our study showed a preference for blood-based kits, which is similar to findings from studies in the USA, Malawi and Zimbabwe [23, 24]. Also our findings of preferences for a low or no cost to the user for HIVST kits, resonate with studies conducted in a wide range of settings (high to low income) and within general and key populations [23, 25, 26]. These characteristics provide an inclusive HIVST program that increases access for testing particularly for those belonging to a low socio-economic group [27, 28].

Similar to other studies conducted in key populations, pharmacies and community distribution were popular choices for preferences of accessing HIVST kits [29–31]. HIVST kits being purchased currently in the Philippines via the internet, without regulation, is not desirable. MSM and TGW were concerned about fake kits being sold, or being discovered by family members on delivery. To optimise access in a highly stigmatised context, multiple avenues of distribution must be considered. Co-ordinated activity between policy makers, regulators and the national laboratory is required and could require policy adaptation to ensure high uptake of kits and testing.

Our results are consistent with studies conducted in key populations, about the importance of receiving counselling for adequate linkage to care [17]. One study in Malawi did report a high degree of linkage, by commencing ART at home [28, 32]. Transferring this model to the Philippines may be difficult, as concerns regarding maintaining confidentiality in the home-environment were raised. Alternative strategies from community based screening that have demonstrated success in a highly stigmatised context could be useful. In China, non- medical outreach workers provided an accompanied referral to MSM to HTS, which increased linkage to care [33, 34]. The Philippines will require an evidence-based intervention to ensure linking these hidden populations to care.

Currently in the Philippines, the UNAIDS reports 32% of individuals with HIV are on anti-retroviral therapy and 82% remain on this 12 months after commencing treatment [2]. Long-term strategies for epidemic control will rely on strengthening the present HIV system in the country. Addressing current weaknesses in the system, such as increasing HIV awareness, linkage to care, ART coverage and preventing loss to follow-up would overall strengthen the HIV care system. This would consequently increase the capacity for implementing HIVST and current testing modalities. A mix of testing strategies will be required to fully curb this advancing HIV epidemic and ensure capturing hidden populations.

In the backdrop of a conservative and highly stigmatised context, civil society advocacy is necessary to drive change and advocate for HIVST. The surge of HIV cases in the Philippines requires actors involved in HIV policy making to be strategic and proactive to change. Concerns of regulation and legislation for HIVST, although important to address, are similar to stakeholders in other contexts, and can hinder a rapid response [35]. As our study shows a theoretical willingness to use HIVST and joins a growing evidence base that HIVST is highly acceptable to MSM and TGW [17, 20]. There is a need to optimise the current policy and regulation for HIVST, foster country ownership and identify regulatory pathways to ensure the wishes of those most at risk of HIV are best served. Policy makers should consider using evidence informed practice, such as work conducted in Africa and Asia in regards to HIVST [23, 32, 36], to build upon, pilot and catalyse a program.

### Limitations

Choosing Metro Manila had the advantage of including key informants involved with national HIV policies and programs. However results cannot be generalized at the national level, as the study team was not able to discuss.

HIVST with local organisations in Cebu and Davao City, where different epidemic drivers are present such as intravenous drug use. The acceptability of HIVST is theoretical in our study, as most participants had not used the test before. Low-income participants were recruited through community organisations and peer educators and therefore may over-represent individuals who are already engaged in services. Similarly MSM and TGW recruited through online dating applications may not be representative of all individuals in these key populations.

## Conclusions

HIVST was overwhelmingly regarded as a strategy that has the potential to increase testing uptake among key populations in the Philippines. The urgency of this HIV epidemic calls for a proactive response from stakeholders. Strategic thinking is required to meet the full potential of HIVST. The Philippines requires innovation, vision and leadership within its health system in order to curb the world’s leading HIV epidemic. It can no longer be business as usual.

## Data Availability

The qualitative data analysed in this study are not available due to the risk of identifying individual participants.

## Abbreviations

FGD: Focus group discussions
HIV: Human immunodeficiency Virus
HIVST: HIV Self-Testing
MSM: Men who have sex with men
TGW: Transgender Women
